# Restorative Justice Education from Intrajudicial Criminal Mediation Associated Factors

**DOI:** 10.3390/ejihpe11030045

**Published:** 2021-06-24

**Authors:** Mercedes Matás Castillo, Inmaculada Méndez, Cecilia Ruiz Esteban, Gloria Soto

**Affiliations:** 1 Department of Evolutionary and Educational Psychology, Faculty of Psychology, Campus Regional Excellence Mare Nostrum, University of Murcia, 30100 Murcia, Spain; mmatascastillo@um.es; 2 Department of Research Methods and Diagnosis in Education, University of Murcia, 30100 Murcia, Spain; gloriasm@um.es

**Keywords:** education, restorative justice, cultural change, violence, intrajudicial mediation, interpersonal relationships, age

## Abstract

The restorative justice (RJ) paradigm requires coherence among legal, justice, and educational systems to promote a culture of restorative dialogue with victims of violence and to reintegrate aggressors into the community. The objective of this study, from an evolutionary social perspective, was to examine criminal mediation files in the archives of the Murcia Intrajudicial Criminal Mediation Service (UMIM), Spain, to see which variables are associated with which types of violence and understand the contents and adoption of agreements. In this study the sociodemographic, procedural, and interpersonal variables of 216 people who used criminal mediation were analysed. The results showed statistically significant differences concerning age, the procedural moment of referral, and the participants’ relationship. The main conclusions are that the youngest group had a more significant number of encounters with physical violence; most agreements occurred in the initial phase of a judicial procedure; and the majority of agreements had moral content regardless of the age of the parties involved. These factors are of interest to the establishment of judicial and educational restorative models.

## 1. Introduction

### 1.1. Concept and Principles of Restorative Justice: Importance of their Inclusion in Formal Justice Systems and Education

The United Nations (UN) describes restorative justice (RJ) [1,2] as an evolving alternative response to formal criminal justice that enables active participation of all the parties involved. It is compatible with laws that guarantee citizens’ rights and it respects the dignity and equality of each person. Furthermore, RJ emphasizes healing by building understanding and harmony among victims, offenders, and communities. This work highlights the value of restorative methodologies by exploring the relationship between RJ and other ways of doing justice.

The current RJ model has its foundation in social justice, respect for human rights [3], and evidence-based results [4,5,6,7,8,9]. RJ’s cultural contribution as a new paradigm in education, conflict management, and the justice system has been incorporated in New Zealand law since 1989, and since then several countries have incorporated it into their practices and laws. RJ is focused on how much damage is repaired instead of how much punishment is inflicted. The forms it can take include mediation, conciliation, circles, group conferences, and sentencing circles [10].

Criminal law operates in instances of legal uncertainty (principles of legality, non-retroactivity, territoriality) or the possibility of abuse (minimal intervention, presumption of innocence, proportionality) [11]. Restorative practices (RPs) consider every human being to be an inherent part of the community. Therefore, in the case of conflict, their aim is to restore the natural balance through the acknowledgement of the truth and the willingness to assume responsibility for reparations while not excluding the individual from society. RPs have been used for classroom management [12,13], in police encounters with young people [10,14], and even with prisoners to foster their interest in studying to improve their employment prospects after leaving prison and avoid relapses [15]. 

When considering RJ, studies stress the need to allow space for cultural and social factors [16] as well as the repeated dissatisfaction and frustration of victims, offenders, families, and legal operators with formal justice [17]. Economic dissatisfaction lies in the limited success in collecting compensation and the time it takes––for example, five years on average in Spain [18]. Delays in the administration of justice lengthen the suffering of victims, which amounts to a denial of adequate judicial protection and the right to due process [19,20]. In this context, the paradigm of humanity [21,22] demands that justice satisfies the needs of all concerned, be they victims or offenders, and opens the way to a restorative methodology to fulfil this requirement [23].

RJ is a social science [24] that offers participants the possibility of experiencing the creation of justice instead of being a passive recipient of it [25]. The benefits derived from this concept include personal, academic, and community results, self-esteem, aspirations for higher studies, feelings of belonging, trust in people in the school environment, and connection with the community [26,27]. In economic terms, UN studies [10,28] argue that the initial cost of community-based programs pays off in the long term by preventing the likelihood of re-offending. In addition, RJ programs are shorter than formal justice processes, thereby reducing institutionalization costs and the burden on overloaded courts. 

The successful implementation of RJ programs requires the support of governments, institutions, legislation, and public education to understand that such programs are not only used in experiments or for specific issues [25,29]. European Directive 2012/29/EU [30] is binding legislation dedicated to RJ. Among the non-binding legislation, Recommendation (2018)8 [23] establishes standards on rights, support, and protection for crime victims.

### 1.2. The Connection between Social, School, and Judicial Violence

According to international data, 32% of students surveyed had suffered bullying by their peers at least once in the previous month. Bullying affects boys and girls equally, and though physical bullying decreases with age, the risk of cyberbullying increases [31]. International organizations and researchers [31,32,33,34] have extensively documented the consequences of violence on young people’s mental health, quality of life, academic performance, future employment, risk behaviors, and adult personality [35,36,37,38,39].

UNESCO’s connection between social and school violence is highlighted internationally [31,32,39] because the countries with the lowest crime rate are also those with the lowest prevalence of school bullying. The treatment of violence perpetrated by children and adolescents varies from country to country in penalties, criminal proceedings, and ages of responsibility. In England, children can be tried at 10 years old, 14 in Italy or Chile, 15 in Sweden, or 16 in Spain [40].

Education can reflect violent attitudes [32], but it can also cushion social or environmental violence and consolidate peace [33]. Therefore, teaching emotional intelligence, empathy, ethical principles, stress management, and interpersonal relationships is essential. Parents, teachers, and headmasters play key roles in this process [41,42,43,44,45,46,47].

To accommodate RJ, we need to start from a multidimensional concept of violence [48,49]. It is therefore necessary to: change attitudes about authority [50], discipline, and responsibility for conflict management [51,52]; include RJ principles in school curricula [53,54]; and develop professional leadership skills [55]. Without this change in thinking, mediation or RPs will in practice be seen as disguised punishments [54].

The concept of norm transgression followed by the imposition of power is still the primary treatment for school violence in Spain [54]. This approach involves inflicting punishment and requires complaints. Prevention attempts are based on control and disciplinary measures. In the case of the Autonomous Community of the Region of Murcia (CARM), for example, the Region’s Coexistence Plan [56] contemplates peer mediation as an alternative, preventive, and residual system. Severe cases of harassment are excluded, and RJ is not mentioned.

In light of this situation, the evidence shows what Anderson [57] calls a “vicious circle of discipline”; that is, the relationship between disciplinary consequences, such as expulsions and suspensions, and academic results, because putting offenders in the juvenile criminal justice system for maintenance of violent behavior [58] can lead to their dropping out of school [59,60].

### 1.3. Experiences with Restorative Justice and Criminal Mediation in Spain

In Spain, the symbiosis between formal justice and restorative dialogue has yet to be developed. According to Giménez-Salinas [61], there is no standard policy for criminal mediation since the practices carried out since 1990 vary according to different autonomous communities (Comunidades Autónomas). Most often it is used for juvenile offenders, under the Organic Law (LO) 5/2000 [62], with principles of judicialization developed by professionals in mediation associations. Comparative studies of pilot experiences in Spain also point to these characteristics [63,64].

Between 2005 and 2008, the General Council of the Judiciary (CGPJ) developed a project with the courts and mediation associations in various parts of Spain to deal with the excessive judicialization of daily life, school disputes, and cases that reach the juvenile prosecutor’s office. The initial question raised was: “[Is it] possible to create an instrument that reduces violence, both interpersonal and that exercised by the penal and penitentiary institution”? [65] (p. 7). Such a system is committed to an intrajudicial model with the collaboration of mediation associations and the judicial referral of cases.

In addition, the Superior Court of Justice (TSJ) of Murcia implemented the New Judicial Office, a new model of organizational structure in shared macroservices consisting of General (SCG), Procedural Order of Procedure (SCOP), and Execution (SCEJ). In this context, the Murcia Intrajudicial Criminal Mediation Service (UMIM) was created at the end of 2013 and integrated into the SCOP. The UMIM includes the novelties of existing within the court system, being directed and served by three officials at judicial headquarters and having the collaboration of certified volunteer mediators [66]. The UMIM provides free mediation services in family, criminal, civil, minor, and contentious administrative areas. In criminal matters involving the Statute of the Victim, LO 4/2015, [67], the UMIM is based on RJ principles and accords with the protocol and operational model of the previous national experience [65,68]. The criminal process continues parallel to mediation, whether the matter is under investigation, prosecution, or execution. If participants reach an agreement, the public prosecutor will take the mediation into account by applying the principle of opportunity, and the judge will obtain some benefit for the offender.

The procedure in criminal mediation [66] begins with contacting the respondent to assess a willingness to engage in a dialogue, and then the mediators contact the complainant. If both parties agree to mediation, they prepare for a meeting that can be held jointly or in separate rooms. The dialogue takes place with the support of mediators who pay special attention to the participants’ needs, emotions, and creative solutions. The adoption of agreements considers both legal viability and adaptation to the particularities of the case within restorative values. In light of the preceding, we propose the following working hypotheses:

**Hypothesis** **1** **(H1).** *Sociodemographic variables are associated with (a) criminal roles (victim or aggressor), (b) the type of violence that motivates mediation, and (c) the content of agreements reached.*

**Hypothesis** **2** **(H2).** *Mediation outcomes (agreements or non-agreements) are associated with a) the time in which the restorative intervention occurs and b) the prior personal relationship of the participants.*

**Hypothesis** **3** **(H3).** *Agreement contents and non-agreement reasons are associated with (a) the participants’ previous personal relationship, (b) the procedural moment of the restorative intervention, and (c) the type of violence.*

This study intends to help elucidate which people’s needs must be considered, both in the selection of, and the approach to, cases of intrajudicial criminal mediation, and when restorative programs in schools to prevent violence should be designed.

## 2. Materials and Methods

### 2.1. Participants

The study participants were 216 users of the Murcia Intrajudicial Mediation Service, who were involved in 88 mediations from 2018 to 2020. The subjects belonged to different social and educational levels, were aged between 18 and 70+ years, and were 59.7% male, 91.2% Spanish, and 8.8% foreign. The number of people participating in each mediation ranged from 2 to 6, with an average of 2.4.

### 2.2. Instruments

The files in the archives of the UMIM for the years 2018–2020 provided the data from which the researchers extracted the following variables: gender (male, female), age (18–39, 40–50, 51 or more years), criminal role (offender, victim or cross-reported), procedural moment of mediation (the start, at trial before sentencing or at the execution after sentencing), previous relationship of the participants (none, economic interest, coexistence or closeness), type of conflict (material, verbal or physical violence), result of mediation (agreement or no agreement), content of the agreement (moral commitment, financial or both), reasons for non-agreement (poor relationship of the parties, economic discrepancies or both).

### 2.3. Procedure

Authorization for access to the archives and judicial files was requested from the Head of Service of the Intrajudicial Mediation Unit and obtained through the Government Secretariat of the Superior Court of Justice of Murcia. Criminal conflicts were selected for this study to represent restorative principles more clearly.

The data collected in the reports of the previous three years were reviewed for relevant variables for possible associations. Mediations that were carried out in 2018, 2019, and 2020 were counted and the files in the archives consulted. To do this, alphanumeric coding of the data of the variables to be studied was carried out, thus maintaining the confidentiality and anonymity of the participants. Mediation users signed, at the time of mediation, the minutes of the information session and the request for service and acknowledged having been informed of the essential characteristics of mediation: confidentiality, voluntariness, good faith, and the mediator’s impartiality and neutrality. They also agreed on the possibility of session viewing for educational purposes and the incorporation of their data in a computer-based dataset.

For the selection criteria, all people involved had to participate in finished mediations regardless of outcome; the data of the target variables had to be obtainable; and all required signatures had to be included in the minutes of both the initial information and final sessions or final agreement.

### 2.4. Analysis of Data

This study followed a non-experimental, cross-sectional design since the three years of data were considered as a whole. The statistical package SPSS (Statistical Package for the Social Sciences), version 24.0 (IBM, Armonk, NY, USA) was used for the statistical analysis. After checking the parametric assumptions, descriptive techniques (frequencies, percentages) and contingency tables with Pearson’s Chi-square test were used to test the variables.

Cramer’s V coefficient was used for Pearson’s Chi-square test tables to determine the effect size. A coefficient of 0.10 was considered low, 0.30 medium, and 0.50 high. The relationships between the independent variables (gender and age), and the dependent variables (criminal role, violence, mediation results, content of agreements, and where appropriate, reasons for no agreement) were studied. The previous relationship of the parties, procedural moment, and type of conflict were studied in relation to the mediation outcomes, the content of the agreements, and the reasons for no agreement.

## 3. Results

### 3.1. Sociodemographic Variables, Conflict, and Content of Agreements

The corrected Chi-Square test suggested that all variables considered were independent of gender: criminal role ($x^{2}$ ^(2)^ = 5.911, *p* = 0.052), type of conflict ($x^{2}$ ^(2)^ = 1.818, *p* = 0.403), mediation outcome ($x^{2}$ ^(1)^ = 0.516, *p* = 0.472), agreement content ($x^{2}$ ^(3)^ = 4.769, *p* = 0.190), and reason for no agreement ($x^{2}$ ^(3)^ = 2.929, *p* = 0.403).

Age (Table 1) was found to be statistically significant in relation to criminal role ($x^{2}$ ^(4)^ = 13.298, *p* = 0.010) with a low effect size (V = 0.177; *p* = 0.009). It is important to note that the youngest age group had the highest number of cross-complaints (62.2%), which were very scarce in the older group (16.2%). However, the unique role, whether victim or offender, was distributed similarly amongst all ages.

Regarding violence, Pearson’s Chi-square test (4) = 22.778, *p* < 0.001) found a statistically significant association with age, with a low effect size (V = 0.226; *p* < 0.001). Conflicts related to a material nature or economic claims were mostly found (44.4%) in the middle-aged group (40–50). Conversely, the youngest group has significantly more conflicts involving physical violence (52.6%) than the other two groups.

The association between mediation outcome and age was found to be non-significant, although the content of the agreements was significant in relation to age ($x^{2}$ ^(6)^ = 23.127, *p* = 0.001), with a low effect size (V = 0.229, *p* = 0.001). Agreements in exclusively economic terms were almost non-existent (0.9%) and all of were in the youngest group. Agreements with exclusively moral content were also mainly found amongst young people (56.6%). In the oldest group, agreements with mixed content (both moral and economic) prevailed. Pearson’s Chi-square test ((6) = 16.967, *p* = 0.009) and Cramer’s V coefficient (V = 0.229; *p* = 0.007) showed a statistically significant association between reasons for no agreement and age with a low effect size. Poor personal relationships combined with financial disagreements were the predominant cause of termination of mediations in the middle-aged group (76.9%). Difficulties derived from exclusively poor personal relationship prevented agreements in young (38.5%), middle-aged (30.8%), and older (30.8%) groups in a similar way. Purely economic reasons were scarce.

### 3.2. Temporal and Personal Variables, Conflict, and Mediation Outcome

Regarding mediation outcome (with or without agreement; Table 2) and previous relationship, a statistically significant association between the variables was found ($x^{2}$ ^(2)^ = 19.071, *p* < 0.001) with a medium effect size (V = 0.271; *p* < 0.001). Most agreements were reached when the participants did not know each other (95.6%) or when they had previous relationships of coexistence or closeness (72.7%). The percentage of agreements (59.0%) and no agreements (41.0%) was similar when the previous relationship was exclusively economic.

The procedural moment in which the mediation took place significantly affected the outcome of the mediation ($x^{2}$ ^(2)^ = 15.016, *p* = 0.001), with a medium effect size (V = 0.280, *p* < 0.001). The majority of referrals arrived at mediation at the beginning of the judicial process, and most ended with an agreement (80.5%). Similarly, 75.0% that were derived after execution reached an agreement. In contrast, while on trial, the percentage of non-agreement (52.9%) was slightly higher than agreement (47.1%).

The type of conflict did not show a significant association with the mediation outcome ($x^{2}$ ^(2)^ = 1.044, *p* = 0.593). For all types of violence, agreements were in the majority.

When the mediation ended in an agreement, its content (Table 3) showed statistically significant associations with a previous relationship of the parties ($x^{2}$ ^(6)^ = 45.169, *p* < 0.001) with a medium size (V = 0.299, *p* < 0.001). Economic agreements alone (0.9% of the total sample) did not occur between strangers or those who had a close relationship, only between people whose relationship was merely contractual or economic. People with close relationships preferred reparations (exclusively moral or personal, 72.4%) or mixed (both moral and economic, 48.8%). Strangers agreed on mixed reparations (42.2% of people with no previous relationship) or exclusively moral (53.3%).

A statistically significant relationship ($x^{2}$ ^(6)^ = 34.371, *p* < 0.001), with a medium effect size (V = 0.263; *p* < 0.001) was found between the procedural moment and the content of the agreements: in execution, the agreement content was mixed in all cases; in prosecution, it was mainly mixed (87.5%); and at the beginning, the vast majority were moral agreements or personal commitments (97.4%).

The type of conflict was significantly associated with the content of the agreements ($x^{2}$ ^(6)^ = 102.292, *p* < 0.001) with a size of medium-high effect (V = 0.442, *p* < 0.001). In material crimes, mixed agreements with both economic and moral reparations were preferred (72.6%). In verbal violence crimes, the percentage of exclusively moral reparations was high (38.2%) and even more in cases of physical violence (59.2%).

In cases where mediation ended without an agreement, we investigated the reasons (Table 4). Significant associations were obtained for the three independent variables: Participants’ previous relationship ($x^{2}$ ^(6)^ = 36.649, *p* < 0.001; V = 0.259; *p* < 0.001; medium effect size), procedural moment of referral (χ2 (6) = 31.489, *p* < 0.001; V = 0.317; *p* < 0.001; medium effect size), and type of conflict ($x^{2}$ ^(6)^ = 29.961, *p* < 0.001; V = 0.241; *p* < 0.001; low effect size).

Most people who did not reach an agreement (71.8%) had a previous relationship of closeness or coexistence (family, friends, neighbors), and disagreement was due to bad personal relations. Economic reasons were scarce and only occurred amongst strangers. Mixed reasons (both economic and personal) were found in mediations in which people had close (61.5%) or contractual (38.5%) relationships, but they did not occur amongst strangers.

For the procedural moment of the derivation, previous poor personal relationship between the parties was found to be the main reason at the beginning (76.9%) and the execution (5.1%) phase. Mixed reasons were the majority in the prosecution phase (69.2%) although exclusively economic and personal reasons were also found.

Lastly, considering the type of conflict, a poor personal relationship prevented agreements in the vast majority of the cases, either exclusively (61.9%) or mixed with economic motives (20.6%).

## 4. Discussion

The data of the present study allowed us to examine associations in criminal mediation processes among gender, age, time mediation, participants’ interpersonal relationships, types of violence, mediation outcomes, contents of the agreements, and reasons for non-agreement.

Gender was not significant in our study regarding criminal role (aggressor or victim) although it had been said to be significant in numerous studies both in judicial [65] and school studies [69]. However, these judicial studies lacked proper statistical analysis and the school studies over-represented male students in all discipline referrals. One explanation for this result may lie in the expressed prohibition of mediation in gender-based violence by Spanish law (art. 87.ter.5 LOPJ) [70]. Another explanation could be that a greater proportion of female aggressors agreed to initiate mediation in Murcia because it was voluntary. A third reason could be that a greater number of males who were initially victims became aggressors, so the initial aggressor was also considered as a victim. This idea may be supported by the large number of cross-reporting among the males observed.

Regarding age, the youngest age group had notably more conflicts with physical violence and cross-complaints than the other two age groups. In contrast, the middle-aged group had more conflicts involving material violence. However, even though the main type of violence was different among the groups, age did not affect the proportion of agreements since mediations mostly ended with agreements in all age categories.

Regarding the procedural moment of intervention, most mediations with agreement took place in the procedural phase, similar to the previous study [65] (p. 141). The procedural moment of the trial (before sentencing) is associated with fewer agreements. In this phase, the parties were less willing to talk due to bad personal relationships or perhaps because of the expectations at trial. Here, the collaboration of lawyers was considered essential for advising their clients in favor of mediation. In the execution of sentence phase, agreements on issues regarding non-payment of pensions stood out. In these cases, mediation may have helped the offender take responsibility and present an alternative to punishment (prison sentence), which usually did not solve the problem for the families.

Regarding social and interpersonal relationships, these results indicated a greater effectiveness in applying restorative principles at the beginning of conflict management. Moreover, these results highlighted the importance of both prevention at the educational level and immediacy at the judicial.

Agreements on moral reparations (apologies, respect, better communication) appeared in all age groups, as in the CGPJ study [65] (p. 143). Such findings emphasize the importance of personal relationships. Personal explanations and compromises that considered the prior reality of the participants would not have been possible at trial. The youngest group mainly included this type of content exclusively even in cases where participants were strangers. In contrast, the oldest group usually mixed moral content and economic reparations. On the other hand, the fact that people had highly negative previous relationships was a fundamental reason for not reaching agreements, which further supported the importance of personal relationships.

These data supported the use of RPs at any age, with the necessary adaptation in objectives and contents. Our results indicated that all people put a higher value on making amends than in the legal classification of the crime. Therefore, reflection was suggested when prosecutors apply the principle of opportunity in court [71]. Moreover, it is crucial to consider this preference when reviewing school coexistence regulations on the treatment of bullying and cyberbullying to open the path to RPs when appropriate.

The limitations of the study include the effect size, which in some associations was low, so a larger sampling size would be needed to verify the results. For example, the study could be expanded to include mediations of previous years. Another limitation was the socioeconomic data from the participants, which prevented us from seeing cultural differences. The vast majority were Spanish citizens and few were from other countries or cultures.

For future studies, it would be interesting to collect data from mediations carried out in schools to review similar variables to establish similarities and differences that reinforce the evolutionary nature of the study. The results of this research encourage broadening the analysis of these variables to the rest of the areas of intrajudicial mediation, especially to consider the specific characteristics of the users in family and civil areas. Such knowledge may help apply restorative methodologies to non-criminal settings, which is already being conducted in educational settings. 

Obtaining qualitative data on the level of user satisfaction with the process is essential to examine likely psychological and cultural changes, such as level of empathy, the concept of authority, and the willingness of participants to advise others to go to mediation.

## 5. Conclusions

Conflicts and violence present diverse manifestations at all ages, with physical violence being more frequent in the youngest people. The moment damage occurs, attention to the case constitutes the first form of reparation by making adequate judicial protection possible. Restorative processes provide a helpful violence prevention mechanism and agile treatment adapted to the needs of victims and aggressors once violence occurs. They also ensure victim reparation, truth, responsibility, and community participation in promoting the positive reintegration of the offender.

In our analysis, we noted that when people have the opportunity to speak and give explanations in a mediation, they mostly reach agreements with moral reparations and commitments to respect and changes attitudes, in addition to economic reparations if applicable. This pattern applies regardless of whether the disputants have a close relationship or do not know each other. Poor personal relationships, however, can certainly affect the flow and outcome of mediations, especially when it comes to close people (family, friends, neighbors). Therefore, to prevent violence, we advocate for relationship care education from the first years of schooling.

The cultural change that would make incorporating RJ into the justice and education systems possible requires an approach open to democratic dialogue and supported by all affected sectors (e.g., families, schools, institutions, police). Having a multidimensional understanding of the conflict and the needs of people [48,72,73] is paramount for ensuring the correct application of restorative methodologies to favor dialogue and the preservation of human dignity.

## Figures and Tables

**Table 1:** Criminal role, type of conflict, outcome, content of agreements, and reasons for the non-agreement of mediation according to age.

Variable	Age			$x^{2}$	*p*
	18–39	40–50	51+		
Criminal role				13.298	0.010
Offender	29 (33.0%)	34 (38.6%)	25 (28.4%)		
Victim	34 (37.4%)	23 (25.3%)	34 (37.4%)		
Cross complaints	23 (62.2%)	8 (21.6%)	6 (16.2%)		
Conflict				22.778	<0.001
Material violence	21 (23.3%)	40 (44.4%)	29 (32.2%)		
Verbal violence	25 (50.0%)	12 (24%)	13 (26.0%)		
Physical violence	40 (52.6%)	13 (17.1%)	23 (30.3%)		
Outcome				3.845	0.146
With agreement	67 (41.4%)	43 (26.5%)	52 (32.1%)		
Without deal	19 (35.2%)	22 (40.7%)	13 (24.1%)		
Agreement content				23.127	0.001
Personal/moral commitment	43 (56.6%)	16 (21.1%)	17 (22.4%)		
Economic	2 (100%)	0 (0.0%)	0 (0.0%)		
Both	22 (26.2%)	27 (32.1%)	35 (41.7%)		
Reason for non-agreement				16.967	0.009
Bad personal relationship	15 (38.5%)	12 (30.8%)	12 (30.8%)		
Economic disagreement	2 (100%)	0 (0.0%)	0 (0.0%)		
Both	2 (15.4%)	10 (76.9%)	1 (7.7%)		

Source: The authors.

**Table 2:** Mediation outcomes based on the previous relationship of the parties, procedural moment of referral, and type of conflict.

Variable	Outcome		$x^{2}$	*p*
	With Agreement	Without Agreement		
Previous relationship			19.071	<0.001
None/slight	43 (95.6%)	2 (4.4%)		
Contractual/economic interest	23 (59.0%)	16 (41.0%)		
Coexistence/closeness	96 (72.7%)	36 (27.3%)		
Procedural moment			15.016	0.001
Start	140 (80.5%)	34 (19.5%)		
Judgment	16 (47.1%)	18 (52.9%)		
Execution	6 (75.0%)	2 (25.0%)		
Type of conflict			1.044	0.593
Material violence	65 (72.2%)	25 (27.8%)		
Verbal violence	37 (74.0%)	13 (26.0%)		
Physical violence	60 (78.9%)	16 (21.1%)		

Source: The authors.

**Table 3:** Content of the agreement, depending on the previous relationship of the parties, the procedural moment of derivation and the type of conflict.

Variable	Agreement Content			$x^{2}$	*p*
	Personal/Moral Commitment	Economic Reparation	Both		
Previous relationship				45.169	<0.001
None/slight	19 (25.0%)	0 (0.0%)	24 (28.6%)		
Contractual/economic interest	2 (2.6%)	2 (100%)	19 (22.6%)		
Coexistence/closeness	55 (72.4%)	0 (0.0%)	41 (48.8%)		
Procedural moment				34.371	<0.001
Start	74 (97.4%)	2 (100%)	64 (76.2%)		
Judgment	2 (2.6%)	0 (0.0%)	14 (16.7%)		
Execution	0 (0.0%)	0 (0.0%)	6 (7.1%)		
Type of conflict				102.292	<0.001
Material violence	2 (2.6%)	2 (100%)	61 (72.6%)		
Verbal violence	29 (38.2%)	0 (0.0%)	8 (9.5%)		
Physical violence	45 (59.2%)	0 (21.1%)	15 (17.9%)		

Source: The authors.

**Table 4:** Reasons for non-agreement, depending on the previous relationship of the parties, the procedural moment of referral, and the type of conflict.

Variable	Reasons for Non-Agreement			χ^2^	*p*
	Poor Relationship	Economic Disagreement	Both		
Previous relationship				36.649	<0.001
None/slight	0 (0.0%)	2 (100%)	0 (0.0%)		
Contractual/economic interest	11 (28.2%)	0 (0.0%)	5 (38.5%)		
Coexistence/closeness	28 (71.8%)	0 (0.0%)	8 (61.5%)		
Procedural moment				31.489	<0.001
Start	30 (76.9%)	0 (0.0%)	4 (30.8%)		
Judgment	7 (17.9%)	2 (100%)	9 (69.2%)		
Execution	2 (5.1%)	0 (0.0%)	0 (0.0%)		
Type of conflict				29.961	<0.001
Material violence	12 (30.8%)	0 (0.0%)	13 (100%)		
Verbal violence	13 (33.3%)	0 (0.0%)	0 (0.0%)		
Physical violence	14 (35.9%)	2 (100%)	0 (0.0%)		

Source: The authors.
